# ETS-NOCV description of chemical bonding: from covalent bonds to non-covalent interactions

**DOI:** 10.1007/s00894-024-06222-6

**Published:** 2024-12-04

**Authors:** Mercedes Kukułka, Olga Żurowska, Mariusz Mitoraj, Artur Michalak

**Affiliations:** 1 Department of Theoretical Chemistry, Faculty of Chemistry, Jagiellonian University, Gronostajowa 2, 30-387, Krakow, Poland; 2 Doctoral School of Exact and Natural Sciences, Jagiellonian University, Łojasiewicza 11, 30-348, Krakow, Poland

**Keywords:** ETS-NOCV, Deformation of molecular electrostatic potential, Chemical bonding, Covalent bonds, Dative bonds, Non-covalent interactions, Polarization

## Abstract

**Context:**

The interpretation of ETS-NOCV for typical covalent and dative-covalent chemical bonds is presented and compared with that for halogen bonds. Possible tuning of the strength of halogen bonding is considered, first by applying an electric field (modeled by the point charges or the electric field vector), and then by constructing a model transition-metal complex with enhanced strength of halogen bonding. For all the systems, the ETS-NOCV picture is supplemented by the analysis of the deformation in molecular electrostatic potential (ΔMEP). The results demonstrate important characteristic features of the analysis based on NOCV: (i) this approach is based on pairs of orbitals with antibonding and bonding character and, thus, allows us to “extract” the “diatomic-like” picture of chemical bonding; (ii) the NOCV-pair contributions to the deformation density often correspond to donation (AB) and back-donation (AB) of electron density between the fragments. The results for halogen bonding demonstrate that it is possible to tune their strength by an electric field in the molecular environment; the halogen-bond energy can reach the order of magnitude typical of dative-covalent bonds. However, the nature of halogen bonds still differs from that of dative-covalent interactions, as the accumulation of electron density between fragments is of significantly lower magnitude. The main effect of the electric field is an increase in the polarization of the fragments, which is clearly manifested by the deformation in the MEP.

**Methods:**

All calculations were performed using the ADF/AMS package. The BLYP exchange–correlation functional was employed with Grimme’s dispersion correction (D3 version) and Becke-Johnson damping, using TZP basis sets. The deformation in the MEP was determined as $\Delta V\left(r\right)={V}^{AB}\left(r\right)- {V}^{A}\left(r\right)- {V}^{B}\left(r\right),$ with the same fragment definition as in the ETS-NOCV method.

## Introduction

Chemical bonding is a fundamental concept in chemistry that underpins our understanding of molecular structure, reactivity, and material properties. For simple diatomic molecules (in particular, homonuclear), the molecular-orbital-based interpretation utilizing the ideas of electron configuration, and the bonding, antibonding, or non-bonding character of orbitals, developed already in the early days of quantum mechanics, [1] provides a clear and educative model that became a standard textbook description in chemistry. For multiatomic systems, however, the delocalized character of molecular orbitals prevents the unique interpretation of the bonding/antibonding character of molecular orbitals for a given bond between any two atoms. Therefore, other approaches are needed. Currently, quantum chemistry offers a variety of quantities and methods suitable for in-depth descriptions of chemical bonding [2–5]. These alternative approaches allow us to understand chemical bonds from different perspectives, each with its own strengths and limitations in terms of accuracy and scaling of computational effort [2–5]. They can be broadly categorized into several groups based on their theoretical foundations.

The first group of methods is based on variationally obtained wavefunctions and covers a wide range of techniques. These include localized bond orbitals [6–11], Valence Bond (VB) methods [12], Domain-Averaged Fermi Holes (DAFH) [13], Localized-orbital Locator (LOL) [14], Conceptual Density Functional Theory descriptors [15–17], the Reaction Fragility Spectra-based approach by Ordon and Komorowski [18, 19], FALDI descriptors by de Lange, Cukrowski et al. [20], quasi-atomic orbitals by Ruedenberg [21, 22] as well as information theory-based quantities developed by Nalewajski within the Information Theory of Molecular Systems [23, 24].

Within molecular orbital (MO)-based approaches, alternative energy decomposition schemes (EDA) are used. They provide physical insight into the nature of a chemical bond by extraction of interaction-energy components (i.e., electrostatic, polarization, etc.). Examples include the Kitaura and Morokuma EDA scheme [25], the related Ziegler and Rauk Extended-Transition-State (ETS) method [26, 27], Head-Gordon’s EDA based on Absolutely Localized Molecular Orbitals (ALMO) [28], the populational space approach by de Silva and Korchowiec [29], Local Energy Decomposition based on CCSD(T) by Neese and coworkers [30], and XEDA energy decomposition by Su [31]. We have also developed a DFT-rooted charge and energy decomposition method, ETS-NOCV [32, 33], by combining the energy decomposition scheme ETS [26, 27] with the Natural Orbitals for Chemical Valence (NOCV) methodology [34, 35]. The latter is in turn based on the Nalewajski-Mrozek bond multiplicity theory [36–41].

The second group of real-space approaches is based on electron density and includes Bader’s Quantum Theory of Atoms in Molecules (QTAIM) [42], Electron Localization Function (ELF) [43, 44], Non-Covalent Interactions (NCI) method based on reduced density gradient [45], Interacting Quantum Atoms (IQA) method [46], its IQA-FAMSEC extension [47], and the Density Overlap Regions Indicator (DORI) [48].

The last, very important group covers perturbation-based energy decomposition methods with the best-known Symmetry-Adapted Perturbation Theory (SAPT) by Jeziorski et al. [49], as well as the Basis Set Superposition Error (BSSE)-corrected scheme by Sokalski and Roszak [50]; it is important to highlight that the latter has been recently further developed in Pernal’s laboratory to tackle excited-state properties [51, 52]. These approaches are designed to accurately describe weak non-covalent interactions in relatively small systems where interaction energies are within a few kcal/mol [49–52].

The ETS-NOCV scheme developed in our laboratory was originally applied predominantly to analyze the metal–ligand bonding in transition metal complexes in the catalytic context and to describe strong covalent bonds [4, 32–35, 53–56]. At a later stage, upon the development of long-range corrected functionals as well as Grimme’s semiempirical corrections [57], ETS-NOCV was proven to be useful and reliable also for analyses of various non-covalent interactions and for describing changes in chemical bonding during chemical reactions [58–64].

In this contribution, we aim to demonstrate the applicability of ETS-NOCV for comparatively describing the transition of the nature of chemical bonds from typical covalent bonds (hundreds of kcal/mol) through dative-covalent connections (dozens of kcal/mol) to a non-covalent regime (where interaction energies are within a few kcal/mol). To illustrate the typical interpretation of NOCV determined for covalent bonds, we discuss the bond between two radicals in CF_3_-CF_3_ and CF_3_-I. Further, borazane (BH_3_-NH_3_) is used as a typical example of a dative-covalent bond. In the central part of the manuscript, we discuss halogen bonding in the NH_3_–-ICF_3_ and NH_3_––ICN systems. We will also consider tuning the strength of halogen bonds in these systems, first by applying an electric field (modeled by the point charges or the electric field vector), and then constructing the model transition-metal complex [Pd(NCI–-NH_3_)_4_]^2+^. The ETS-NOCV picture will be supplemented by the analysis of the deformation in molecular electrostatic potential (ΔMEP), which was recently shown [65] to be useful in a description of chemical bonding.

## Theoretical background

The deformation density, $\Delta \rho \left(r\right)= {\rho }^{AB}\left(r\right)-{\rho }^{A}\left(r\right){-\rho }^{B}\left(r\right)$, corresponds to the difference in the electron density of the molecular system *AB* (${\rho }^{AB}\left(r\right))$ and that of a *promolecule* built of isolated fragments *A* and *B* (in the geometry of *AB*), ${\rho }^{0}\left(r\right)={\rho }^{A}\left(r\right){+\rho }^{B}\left(r\right)$. Here, we assume that only two molecular fragments are considered. The main idea of the *Natural Orbitals for Chemical Valence* (NOCV) method [34, 35] was to provide an orbital set ($\psi$) that allows for its diagonal representation (Eq. 1):

1 $$\Delta \rho \left(r\right)=\sum_{i}^{n}{\nu }_{i}{\psi }_{i}^{2}(r)$$

This can be obtained by diagonalization of the **ΔP = P – P**^**0**^ matrix, which corresponds to the difference in the *charge and bond-order* matrix in the molecule (**P**) and promolecule (**P**^**0**^), expressed in basis set of atomic orbitals (or the Kohn–Sham fragment orbitals) $\varphi$. Thus, the NOCV orbitals are eigenvectors of **ΔP** ﻿(Eq. 1):

2 $${\varvec{\Delta}}\mathbf{P}{{\varvec{\psi}}}_{{\varvec{i}}}={\nu }_{i}{{\varvec{\psi}}}_{{\varvec{i}}}; {\ \ \ } i=1,\dots , n$$

The original name NOCV refers to the relation of **ΔP** to the matrix representation of the total valence operator from the Nalewajski and Mrozek theory of chemical valence and bond-order indices [36–41]; thus, the NOCV orbitals can be considered as the eigenfunctions of this operator.

Furthermore, since the deformation density is normalized to zero and NOCV are orthonormal, they can be coupled in pairs of complementary eigenvectors corresponding to the same absolute value and opposite signs of the eigenvalues ${\nu }_{\pm k}$ [34, 35, 66, 67] (Eq. 3):

3 $${\varvec{\Delta}}\mathbf{P}{{\varvec{\psi}}}_{{\varvec{i}}}={\nu }_{k}{{\varvec{\psi}}}_{{\varvec{k}}} ;{\ \ \ }{\varvec{\Delta}}\mathbf{P}{{\varvec{\psi}}}_{-{\varvec{k}}}={\nu }_{-k}{{\varvec{\psi}}}_{-{\varvec{k}}}; {\ \ \ }k=1,\dots , n/2$$

Finally, the deformation density can be decomposed into the contributions from such pairs of NOCV, ${\Delta \rho }_{k}\left(r\right)$ (Eq. 4):

4 $$\Delta \rho \left(r\right)=\sum_{k}^{n/2}{\nu }_{k}\left[{\psi }_{k}^{2}\left(r\right)-{\psi }_{-k}^{2}\left(r\right)\right]=\sum_{k}^{n/2}\Delta {\rho }_{k}(r)$$

It is essential that only a few eigenvalues are significant, the vast majority of them are close to zero; thus, there are only a few non-negligible NOCV-pair contributions to $\Delta \rho \left(r\right)$. Within those pairs ${\psi }_{-k}$ and ${\psi }_{k}$ usually exhibit antibonding and bonding character, respectively. It should be emphasized that ${\nu }_{\pm k}$ correspond to the changes in the (fractional) electron populations of the two orbitals in the molecule and promolecule, i.e., the charge flow from bonding and antibonding NOCV accompanying the bond formation process [32, 34, 35]. Also, the dominating NOCV-pair contributions, ${\Delta \rho }_{k}\left(r\right),$ describe easily interpreted, meaningful components of chemical bonds (e.g., donation/back-bonding, σ, π, or δ) [61, 62, 68].

Finally, when the open-shell fragments are considered, spin-resolved ETS-NOCV analysis is used, i.e., the spin-α and spin-β NOCV $\left\{\psi_i^\sigma;\;i=1,n;\;\sigma=\alpha,\beta\right\}$ are obtained from separate diagonalizations of **ΔP**^**α**^and **ΔP**^**β**^ matrices, and thus, the deformation-density decomposition of Eq. 4 takes the following form (Eq. 5)

5 $$\Delta \rho \left(r\right)=\sum_{\sigma =\alpha ,\beta }^{\nu}\sum_{k}^{n/2}{\nu }_{k}^{\sigma }\left\{{\left[{\psi }_{k}^{\sigma }(r)\right]}^{2}-{\left[{\psi }_{-k}^{\sigma }(r)\right]}^{2}\right\}=\sum_{\sigma =\alpha ,\beta }^{\nu}\sum_{k}^{n/2}{\Delta \rho }_{k}^{\sigma }(r)=\sum_{k}^{n/2}\Delta {\rho }_{k}(r)$$

In the ETS-NOCV (or EDA-NOCV) method [32], the NOCV approach is combined with the Ziegler-Rauk extended transition state (ETS) energy decomposition analysis (EDA) method [26, 27], where the interaction (bonding) energy of a bond *A-B* between two fragments *A* and *B* is decomposed into electrostatic (${\Delta E}_{elstat}$), Pauli repulsion (${\Delta E}_{Pauli}$), and orbital interaction (${\Delta E}_{orb}$) components (Eq. 6):

6 $$\Delta E_{int}=\Delta E_{elstat}+\Delta E_{Pauli}+\Delta E_{orb}+\Delta E_{disp}$$

The first term, ${\Delta E}_{elstat}$, corresponds to the classical electrostatic interaction between the frozen fragments (in the geometry of the whole molecular system considered), and ${\Delta E}_{Pauli}$ is the repulsive interaction between occupied orbitals of the two fragments; it is common to combine ${\Delta E}_{elstat}$ and ${\Delta E}_{Pauli}$ into the steric interaction energy (${\Delta E}_{steric}= {\Delta E}_{elstat}+{\Delta E}_{Pauli}$). The orbital-interaction term is the stabilizing component due to bond formation, i.e., charge flows from the occupied orbitals to virtual orbitals of the fragments (with mutually orthogonalized orbitals). The orbital interaction term of ETS depends on $\Delta \rho$ which allows for its further decomposition into the contributions from NOCV-pairs (Eq. 7)

7 $${\Delta E}_{orb}= \sum_{k}^{n/2}{\Delta E}_{orb, k}$$

It should be emphasized that following the original Morokuma picture of bond formation in energy-decomposition approaches [25], the orbital-interaction term includes intra-fragment polarizations (mixing of occupied and virtual orbitals of the same fragment) and charge transfer between the fragments (charge flows from occupied orbitals of one fragment to virtual orbitals of the other fragment and vice versa). It is well known that the Morokuma picture is just a model, and the polarization and charge transfer cannot be separated exactly within the incomplete basis sets. On the other hand, it should also be emphasized that in the implementation of the ETS-NOCV analysis, the basis set used in the molecular calculations is composed of the occupied and virtual, variational Kohn–Sham orbitals (still incomplete) of the fragments, and not the atomic basis functions. Therefore, the Morokuma picture can be considered a very useful model; it is particularly valid when the NOCV-contributions corresponding to large eigenvalues (and large energies) are considered (e.g., in the case of strong covalent and typical dative-covalent bonds). Besides Morokuma picture, similar charge-transfer processes between fragments form the basis of the Dewar-Chatt-Duncanson (DCD) model [69, 70], which is very popular in a description of bonding in organometallics, in which the ligand →  metal donation and the metal →  ligand back-bonding are considered. For small-eigenvalue (and small-energy) NOCV contributions (as in the case of non-covalent interactions), the validity of the Morokuma and the Dewar-Chatt-Duncanson model becomes obviously disputable.

It should also be added that only the first three terms in Eq. 6 were considered in the original Ziegler-Rauk ETS analysis. In the dispersion-corrected DFT calculations, an additional dispersion term (${\Delta E}_{disp}$) is used. Furthermore, Eq. 6 defines the interaction energy between molecular fragments in their distorted geometries (as in the whole molecular system considered). Thus, when addressing the bond energy defined as the negative energy of dissociation into fragments, an additional term corresponding to the energy change due to geometry preparation/distortion must be considered.

Recently [65], we proposed supplementing the ETS-NOCV approach by considering the deformation in the molecular electrostatic potential (differential molecular electrostatic potential, ΔMEP) due to bonding between the fragments A and B, defined as in ETS-NOCV (i.e., molecule vs. promolecule, Eq. 8)):

8 $$\Delta V\left(r\right)={V}^{AB}\left(r\right)- {V}^{A}\left(r\right)- {V}^{B}\left(r\right)$$

where ${V}^{AB}\left(r\right), {V}^{A}\left(r\right)$ and ${V}^{B}\left(r\right)$ represent the molecular electrostatic potential (MEP) of the molecular system and fragments *A* and *B*, respectively.

It should be noted that each MEP, ${V}^{AB}\left(r\right), {V}^{A}\left(r\right)$, and ${V}^{B}\left(r\right)$ contains contributions from nuclei and electrons, e.g. (Eq. 9),

9 $${V}^{AB}\left(r\right)= \sum_{j}\frac{{Z}_{j}}{|{R}_{j}-r|}- \int \frac{{\rho }^{AB}({r}{\prime})}{|{r}{\prime}-r|}{d}^{3}r{\prime}$$

where *Z*_*j*_ is the charge of nucleus *j* located at *R*_*j*_. The deformation in the MEP includes only the electronic part (Eq. 10),

10 $$\Delta V\left(r\right)= - \int \frac{\Delta \rho ({r}{\prime})}{|{r}{\prime}-r|}{d}^{3}r{\prime}$$

since the atomic positions are the same in both, the molecular system and the corresponding promolecule.

## Computational details

All the calculations were performed using the Amsterdam Density Functional (ADF) package (version 2017.103) [68, 71, 72]. The BLYP exchange–correlation functional was used [73, 74], coupled with Grimme’s dispersion correction (D3 version) [57, 75] with Becke-Johnson damping [76, 77]. For the non-covalent interactions, the dispersion-corrected BLYP functional was shown to lead to the best agreement between the ETS energy terms and the corresponding SAPT terms [78, 79]. The triple-ζ basis set with one set of polarization functions (TZP) was used [80, 81]. Scalar relativistic corrections were included within the zero-order regular approximation (ZORA) [66, 67, 82]. The studies concerning the effect of the external electric field on the strength of halogen bonds were performed based on a sequence of single-point calculations (for geometries optimized without the electric field), with the electric field modeled by (i) positive point charge and (ii) the electric field vector considered explicitly. The details concerning the models used will be presented together with the results in the “Tuning the strength of halogen bonds” section.

## Results and discussion

### Covalent bond in CF_3_-CF_3_

**Fig. 1:**
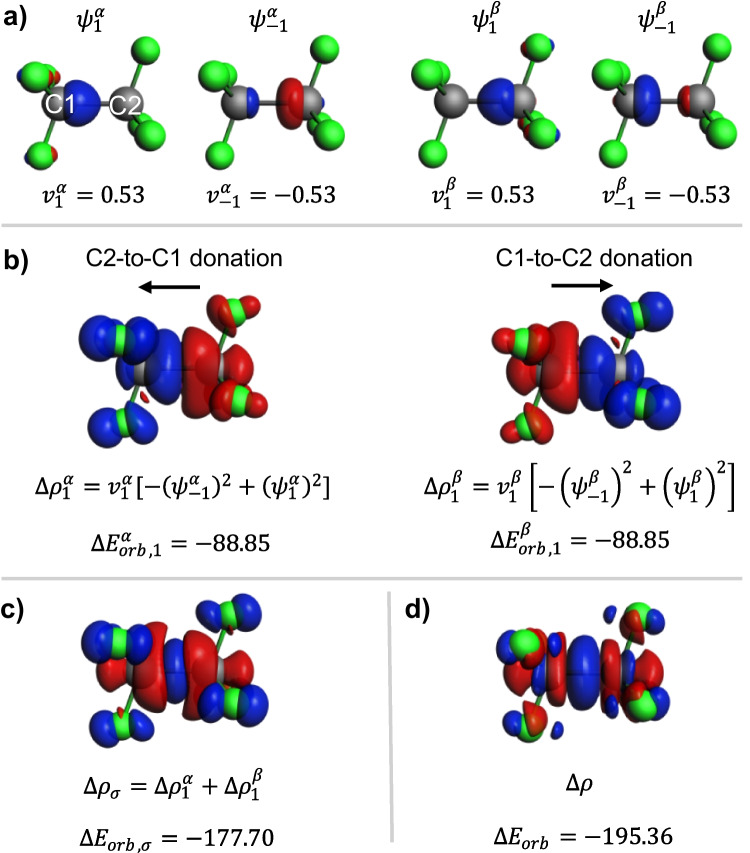
ETS-NOCV description of the σ-symmetry components of the C–C bond in hexafluoroethane and the corresponding deformation density. **a** The dominant spin-resolved pairs of NOCVs along with the NOCV eigenvalues. **b** The corresponding NOCV-pair contributions to the deformation density along with the orbital interaction energies (in kcal/mol). **c** The deformation-density σ-contribution resulting from the two NOCV pairs displayed in panel (**a**). **d** The total deformation density. The isosurface values are |0.2| a.u. for orbitals and |0.002| a.u. for $\Delta \rho$. The blue/red regions of $\Delta \rho$ indicate the accumulation/depletion of the electron density

**Fig. 2:**
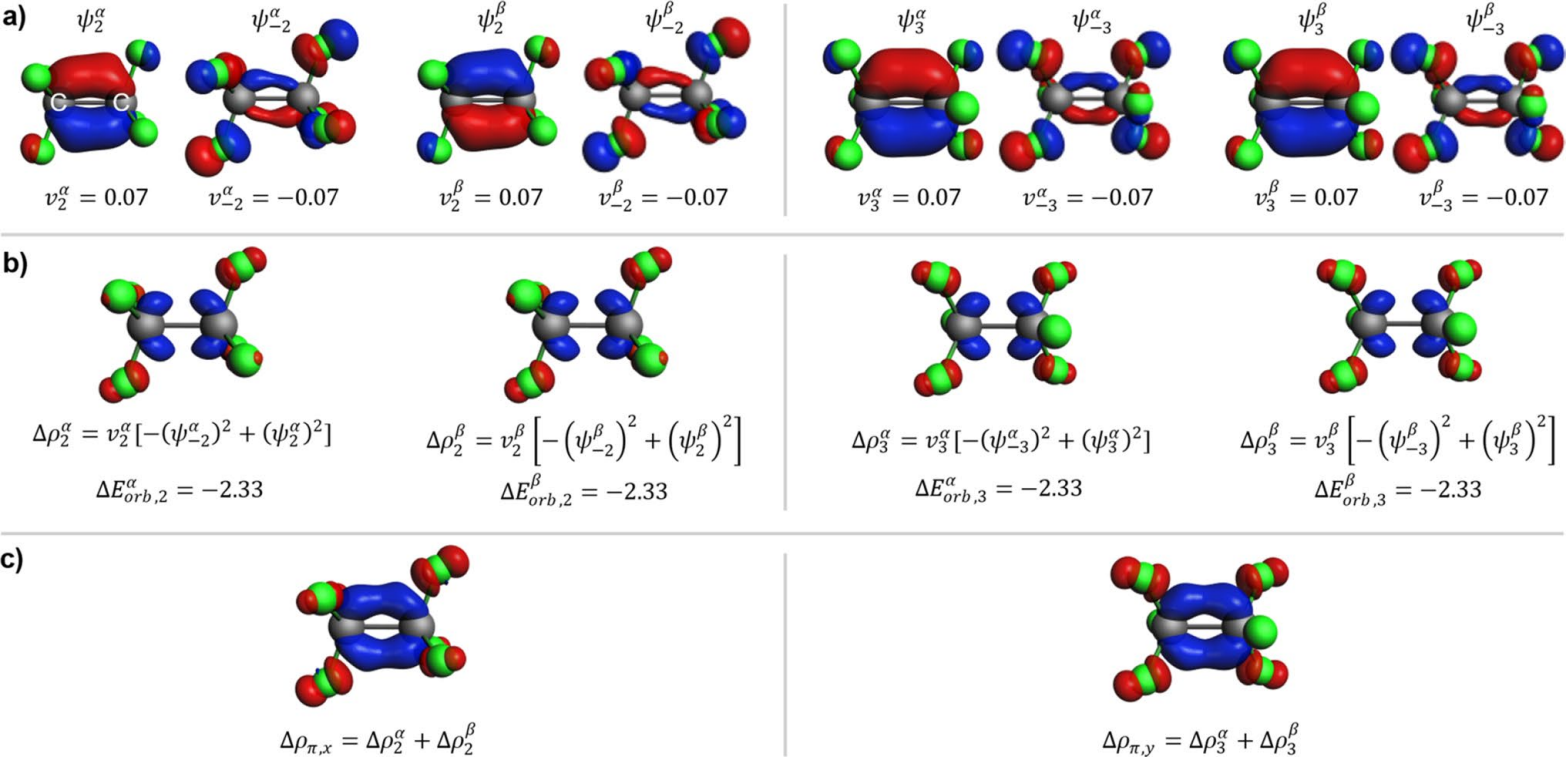
ETS-NOCV description of the π-symmetry components of the C–C bond in hexafluoroethane and the corresponding deformation density. **a** The dominant spin-resolved pairs of NOCVs along with the NOCV eigenvalues. **b** The corresponding NOCV-pair contributions to the deformation density along with the orbital interaction energies (in kcal/mol). **c** The two deformation-density π-contributions, each resulting from one of the two NOCV pairs displayed in panel (**a**). Isosurface values are |0.08| a.u. for orbitals and |0.002| a.u. for $\Delta \rho$. The blue/red regions of $\Delta \rho$ indicate the accumulation/depletion of the electron density

For the sake of presenting a typical interpretation of NOCV, first, we discuss a bond in CF_3_-CF_3_ between two radical CF_3_ fragments. Here, the spin-resolved approach is used (Eq. 5), and the results are summarized in Figs. 1 and 2. Part a of Fig. 1 displays two pairs of complementary spin-resolved NOCV orbitals with dominant eigenvalues ($\left|v\right|=0.53$ a.u.). These orbitals exhibit σ-symmetry, with those corresponding to positive (negative) eigenvalues indicating bonding (antibonding) character. Due to molecular symmetry, the orbitals of spin *α* and spin *β* are related by an inversion center transformation. The corresponding σ-deformation densities (Fig. 1b), $\Delta {\rho }_{1}^{\alpha }=-0.53{\left({\psi }_{-1}^{\alpha }\right)}^{2}+0.53{\left({\psi }_{1}^{\alpha }\right)}^{2}$ and $\Delta {\rho }_{1}^{\beta }=-0.53{\left({\psi }_{-1}^{\beta }\right)}^{2}+0.53{\left({\psi }_{1}^{\beta }\right)}^{2}$, illustrate the electron density shift between the CF_3_ radicals in both directions. This picture indicates an important, intrinsic feature of NOCV analysis: the donation/back-bonding picture of the Dewar-Chatt-Duncanson model. Each $\Delta {\rho }_{1}^{s}$ ($s\in \{\alpha ,\beta \}$) contributes to the orbital stabilization energy, with $\Delta {E}_{orb,1}^{\alpha }=\Delta {E}_{orb,1}^{\beta }=-88.85$ kcal/mol. Combined together $\Delta {\rho }_{\sigma }=\Delta {\rho }_{1}^{\alpha }+\Delta {\rho }_{1}^{\beta }$, as shown in Fig. 1c, these densities depict typical σ-bond formation with charge depletion in the atomic regions and charge accumulation in the bond area. The orbital interaction energies (summed for both spins, $\Delta {E}_{orb,1}=-177.7$ kcal/mol) highlight $\Delta {\rho }_{\sigma }$ as the primary component of the total deformation density $\Delta \rho$ (Fig. 1d), contributing approximately 91% to the total orbital interaction energy ($\Delta {E}_{orb}=-195.4$ kcal/mol). Additionally, two more stabilizing orbital interactions of π-symmetry have non-negligible contributions to the total $\Delta {E}_{orb}$ ($\Delta {E}_{orb,2}$ and $\Delta {E}_{orb,3}$, each with a combined value for both spins of 4.66 kcal/mol), describing the hyperconjugation type of electron density shifts σ(C-F) → σ*(C-F) (Fig. 2).

**Fig. 3:**
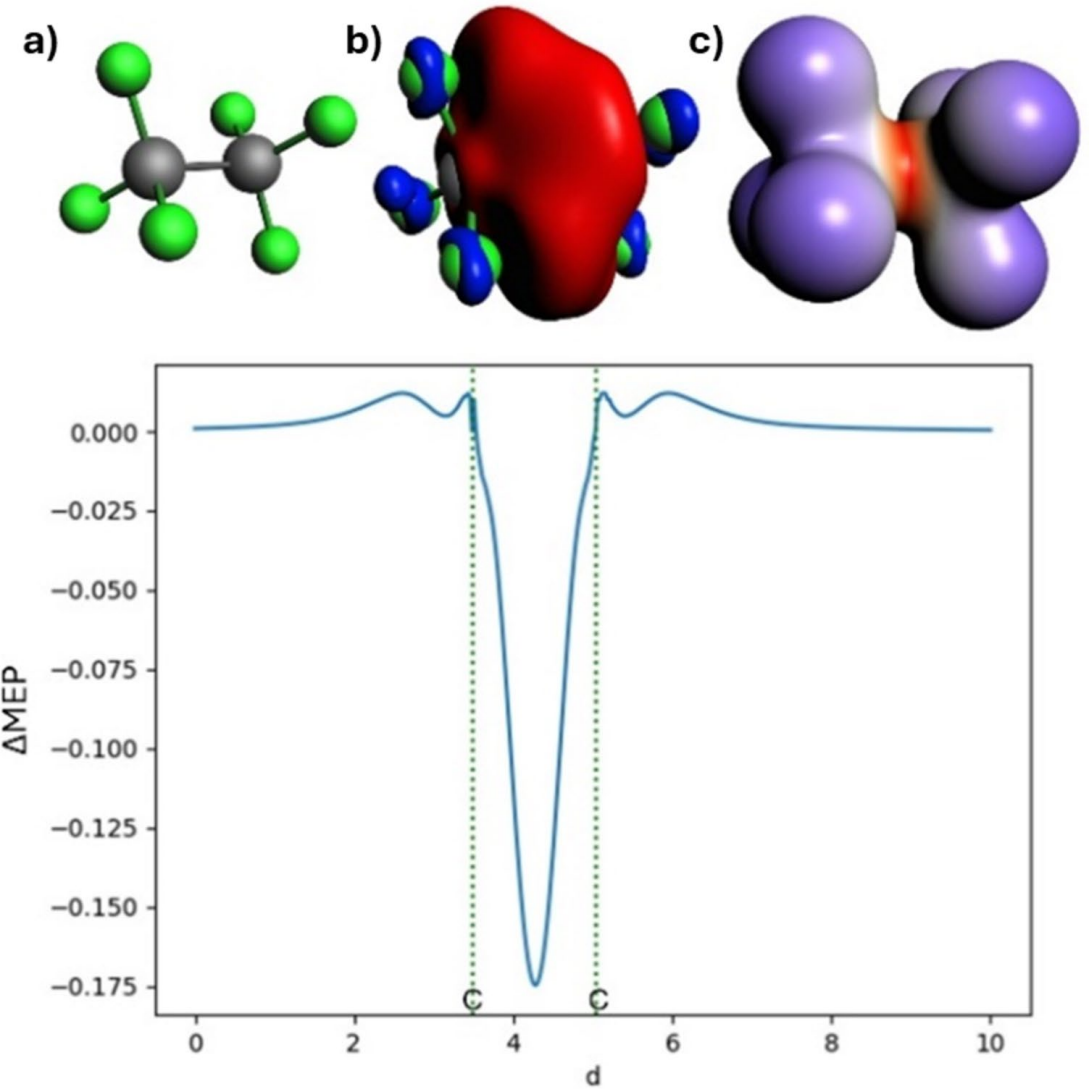
Analysis of ΔMEP for hexafluoroethane: In the top row, molecule (**a**), the ΔMEP contour with a value of 0.01 a.u. (**b**), and the ΔMEP projected onto an isodensity contour of electron density with a value of 0.01 a.u. (**c**) are shown. The positive/negative ΔMEP values are indicated in blue/red. The graph depicts the ΔMEP value along the bond axis of the molecule. The dotted green lines indicate the positions of the atomic nuclei

In Fig. 3, the deformation in the MEP is presented, calculated with the same fragments as in ETS-NOCV; three different graphical representations of ΔMEP are shown: its isocontour, the electron density contour colored according to the ΔMEP values, and the plot of its values along the bond. The image obtained from the differential molecular electrostatic potential is fully consistent with the ETS-NOCV results. As depicted in Fig. 3, the deformation of the molecular electrostatic potential reflects an accumulation of electron density in the C–C bonding region between the atoms (negative ΔMEP) and a density depletion along the bond axis outside the bonding region and on the fluorine atoms (positive ΔMEP). This is clearly observed in all the graphical representations of ΔMEP presented. The minimum ΔMEP value for the C–C bond is reached at a value of − 0.175 a.u.

### Polarized bond in I-CF_3_

**Fig. 4:**
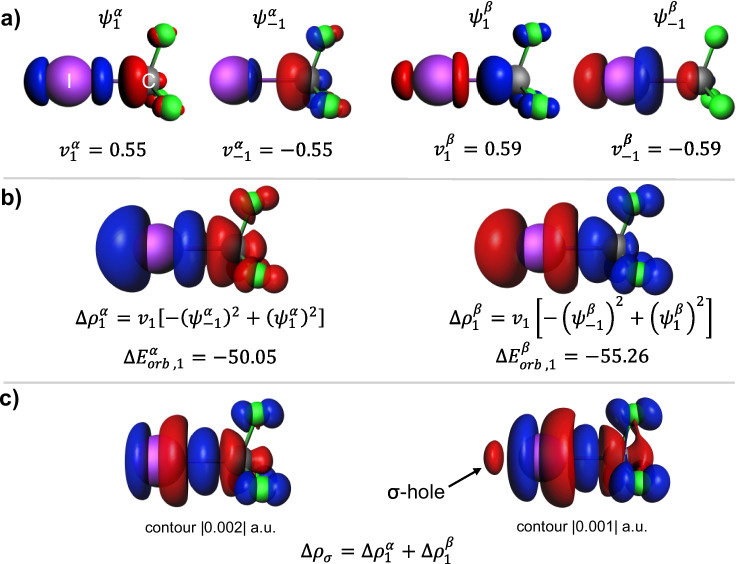
ETS-NOCV description of the σ-hole-bonding components, the C–I bond in trifluoroiodomethane, and the corresponding deformation density. **a** The dominant spin-resolved pairs of NOCVs along with the NOCV eigenvalues. **b** The corresponding NOCV-pair contributions to the deformation density along with the orbital interaction energies (in kcal/mol). **c** The total deformation density. The isosurface values are |0.01| a.u. for orbitals and |0.002| a.u. for $\Delta {\rho }_{1}$; for $\Delta \rho$, two contours are displayed to show the σ-hole. The blue/red regions of $\Delta \rho$ indicate the accumulation/depletion of the electron density

We now briefly characterize the polarized covalent bond between the iodine atom and the CF_3_ fragment in I–CF_3_. Like in the picture of covalent bonds in hexafluoroethane, the NOCV orbitals (Fig. 4a) corresponding to negative/positive eigenvalue exhibit antibonding/bonding character. The spin-resolved deformation-density contributions (Fig. 4b) correspond to the donation/back-donation picture, and the total $\Delta {\rho }_{\sigma }$(summed for both spins, Fig. 4c) indicates the formation of a polarized σ bond, as was shown previously [60, 63]. The corresponding orbital interaction energy (summed for both spins) is $\Delta {E}_{orb,1}=-105.31$ kcal/mol. Please note that the numerical values in the ETS-NOCV results differ slightly from those presented previously [60, 63] as different exchange–correlation functional (BLYP instead of BP86) are used in this study. We would like to emphasize that the important feature of $\Delta {\rho }_{\sigma }$ is an outflow of electron density from the area extending outward from the iodine atom along the axis of the I–C bond, which describes the formation of a σ-hole (Fig. 4c) [60, 63].

**Fig. 5:**
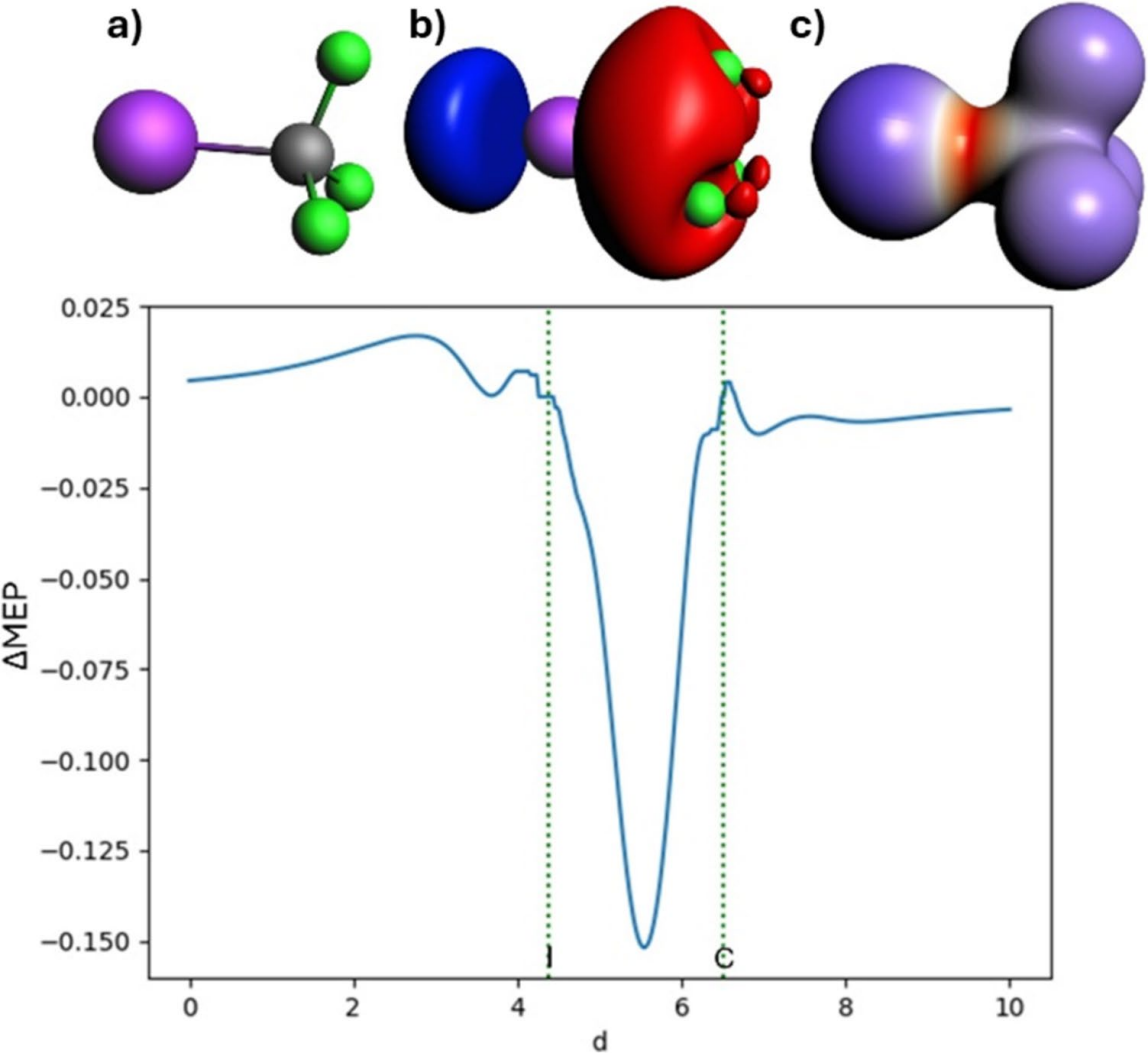
Analysis of ΔMEP for trifluoromethyl iodide: In the top row, the molecule (**a**), the ΔMEP contour with a value of 0.01 a.u. (**b**), and the ΔMEP projected onto an isodensity contour of electron density with a value of 0.01 a.u. (**c**) are shown. The positive/negative ΔMEP values are indicated in blue/red. The graph depicts the ΔMEP value along the bond axis of the molecule. The dotted green lines indicate the positions of the atomic nuclei

In Fig. 5, the corresponding deformation in the MEP is presented. Again, the overall picture is consistent with the ETS-NOCV analysis. The polarized character of the I-CF3 bond is nicely emphasized by the asymmetry of the ΔMEP plots. The minimum between iodine and carbon (− 0.152 a.u.) is also shifted towards the carbon atom A prominent blue area is distinctly visible near iodine at the extension of the bond, indicating the sigma hole. It may be concluded from a comparison of the deformation density and differential MEP pictures that the latter more clearly emphasizes polarization of the fragments (“charge shift”) due to the long-distance character of the electrostatic potential.

### Donor–acceptor bond in borazane

**Fig. 6:**
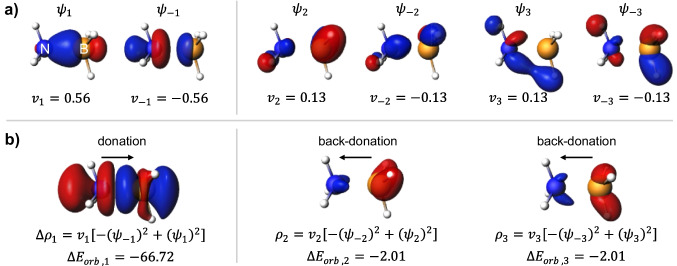
ETS-NOCV description of the σ-symmetry (left) and π-symmetry (right) components in the dative B–N bond in borazane, along with the corresponding deformation density. **a** The dominant pairs of NOCVs along with the NOCV eigenvalues. **b** The corresponding NOCV-pair contributions to the deformation density along with the orbital interaction energies (in kcal/mol). The isosurface values are |0.1| a.u. for orbitals and |0.002| a.u. for $\Delta \rho$. The blue/red regions of $\Delta \rho$ indicate the accumulation/depletion of the electron density

In Fig. 6, the results of NOCV analysis of the textbook example of the dative-covalent B–N bond in the borazane molecule are presented, with a promolecule constructed of ammonia and borane fragments. Although the interpretation of NOCV for borazane was examined in earlier papers [66, 67], we include it here as an example of a typical donor–acceptor interaction. The dominant deformation density channel, $\Delta {\rho }_{1}$, originates from the electron density shift from the lone-pair orbital of ammonia towards borane fragment (mostly the empty 2*p* boron orbital). This charge transfer corresponds to the energetic stabilization, $\Delta {E}_{orb,1}=-66.7$ kcal/mol. The two quantitatively less significant contributions to the B–N bond, $\Delta {\rho }_{2}$ and $\Delta {\rho }_{3}$, with corresponding energies $\Delta {E}_{orb,2}=\Delta {E}_{orb,3}=-2.0$ kcal/mol, characterize π-type back-donation channels, i.e., density outflow from the occupied σ(B–H) orbitals of borane to σ*(N–H) of ammonia.

**Fig. 7:**
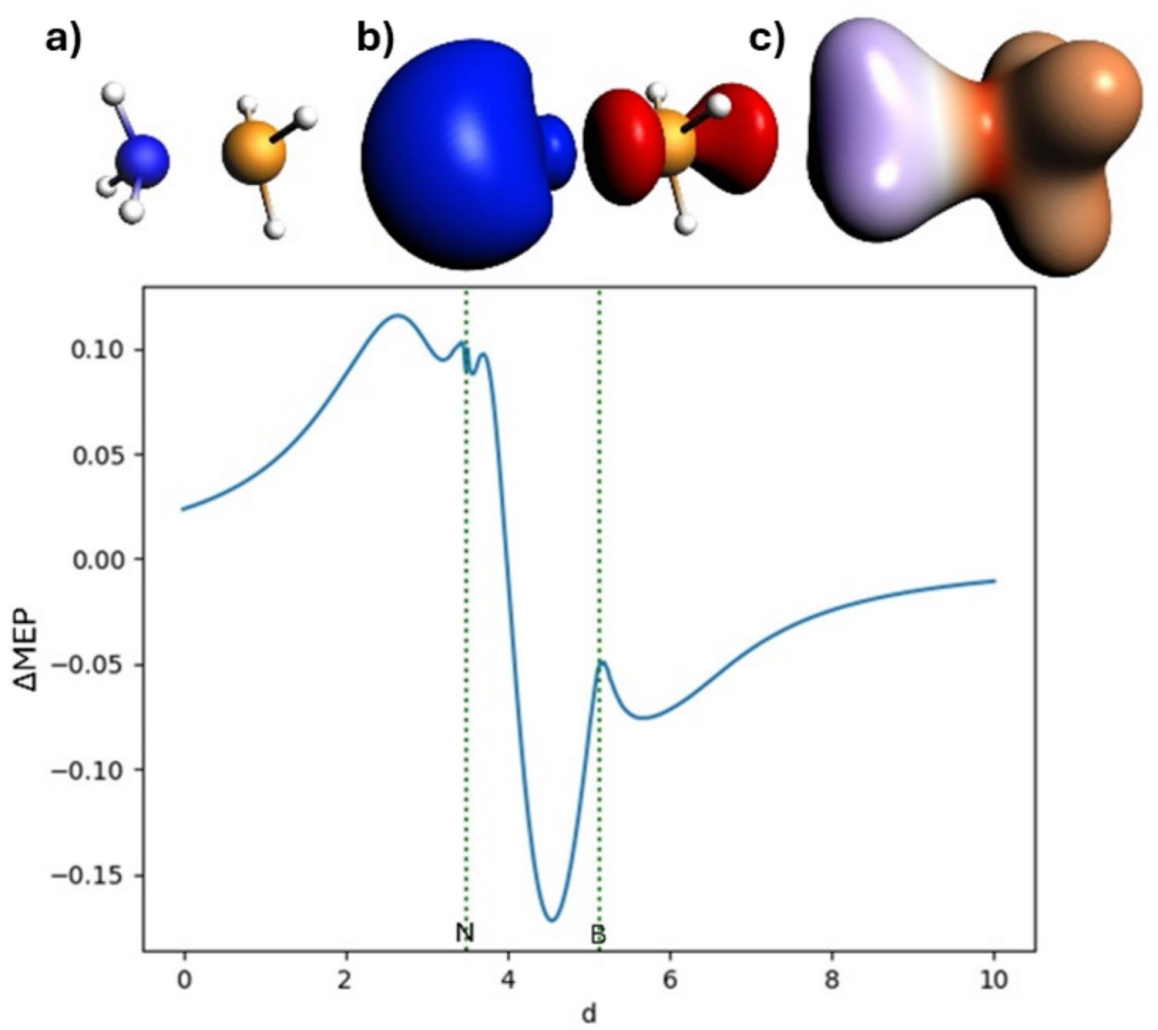
Analysis of ΔMEP for borazane: In the top row, the molecule (**a**), the ΔMEP contour with a value of 0.05 a.u. (**b**), and the ΔMEP projected onto an isodensity contour of electron density with a value of 0.01 a.u. (**c**) are shown. Positive/negative ΔMEP values are indicated in blue/red. The graph depicts the ΔMEP value along the bond axis of the molecule. The dotted green lines indicate the positions of the atomic nuclei

The deformation in the MEP for borazane is illustrated in Fig. 7. As we discussed in the previous case, the ΔMEP picture of the BH_3_-NH_3_ bond is dominated by the NH_3_ → BH_3_ charge shift between the fragments: in the “ammonia area,” the ΔMEP is positive, and in the “borane area,” it is negative. The covalent character of the bond is indicated by the deep (− 0.172 a.u.) minimum of ΔMEP in the bonding region, asymmetrically displaced closer to the boron atom. It is worth emphasizing that this minimum is as deep as that in CF_3_-CF_3_ and in fact deeper than that in I-CF_3_.

### ETS-NOCV description of halogen bonding in NH_3_⋅⋅⋅ICF_3_ and NH_3_⋅⋅⋅ICN

**Fig. 8:**
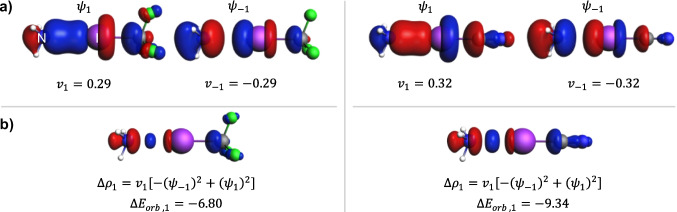
ETS-NOCV description of the σ-symmetry components of the N⋅⋅⋅I interaction in NH_3_⋅⋅⋅ICF_3_ (left) and NH_3_⋅⋅⋅ICN (right) and the corresponding deformation density. **a** The dominant pairs of NOCVs along with the NOCV eigenvalues. **b** The corresponding NOCV-pair contributions to the deformation density along with the orbital interaction energies (in kcal/mol). The isosurface values are |0.05| a.u. for orbitals and |0.002| a.u. for $\Delta {\rho }_{1}$. The blue/red regions of $\Delta \rho$ indicate the accumulation/depletion of the electron density

Building on the NOCV-based description of a σ-hole in I–CF_3_, we will examine the formation of halogen bonds in the NH_3_⋅⋅⋅ICF_3_ and NH_3_⋅⋅⋅ICN systems. The ETS-NOCV description of halogen bonding in the former system was presented previously [60, 63]; however, only the deformation-density dominating contribution was shown. To present the complete interpretation, we include the NOCV orbitals for both systems, as shown in Fig. 8a. As is clearly observed, the NOCV description of halogen bonding is based on a pair of orbitals with bonding and antibonding character. This result is similar to that of the systems with covalent and dative bonds, as discussed above. Furthermore, the *shape* of the deformation density contributions shown in Fig. 8b looks like the typical shape of the σ -bonding orbital density. In particular, there are a lot of similarities in the shape of the deformation density in borazane (Fig. 6b) and in the two halogen bonds discussed here (Fig. 8b). In terms of the Dewar-Chatt-Duncanson and Morokuma models, the deformation density contributions show the “charge transfer” between the ammonia and ICF_3_/ICN. This is the reason why we spoke about “covalent” contributions and “charge transfer” in previous papers [60, 63]. However, one has to remember about the important differences between the NOCV-based picture of covalent/dative bonds discussed in the previous sections and the halogen bonds discussed here. For halogen bonds, the NOCV eigenvalues are relatively small; thus, both antibonding and bonding NOCV have fractional occupation. Furthermore, the values of the orbital interaction energy are relatively small for both NH_3_⋅⋅⋅ICF_3_ and NH_3_⋅⋅⋅ICN ($-6.8$ and $-9.3$ kcal/mol, respectively). Indeed, the complete set of ETS-NOCV results, summarized in Table 1, shows that the magnitude of the electrostatic energy is approximately twice that of the orbital interaction term; thus, it is the major contribution to system stabilization. For comparison, Table 1 also shows that in the case of covalent bonds discussed here, the magnitude of the orbital-interaction term is much greater than that of the electrostatic term and in the case of borazane dative bond. We would like to emphasize that we have concluded a previous paper [63] by saying that “the results of the present analysis confirm the indisputable role of the electrostatic stabilization in halogen bonding and σ-hole bonding, emphasized in the previous articles by Politzer and coworkers.”

**Table 1:** The ETS-NOCV interaction energy components as defined in Eq. 6 (in kcal/mol). Δ*E*_orb,1_ corresponds to the dominant NOCV-pair contribution to the orbital interaction energy (see Eq. 7). The red vertical line indicates the fragments considered

System	Δ*E*_int_	Δ*E*_disp_	Δ*E*_elstat_	Δ*E*_Pauli_	Δ*E*_steric_	Δ*E*_orb_	Δ*E*_orb,1_
CF_3_–**|**CF_3_	− 92.85	− 2.83	− 141.49	246.83	105.34	− 195.36	− 177.7
I–**|**CF_3_	− 58.22	− 3.12	− 85.24	147.57	62.33	− 117.43	− 105.31
NH_3_–**|**BH_3_	− 42.10	− 2.32	− 75.50	108.21	32.70	− 72.48	− 63.39
NH_3_⋅⋅⋅**|**ICN	− 10.78	− 2.38	− 20.51	22.64	2.13	− 10.52	− 9.34
NH_3_⋅⋅⋅**|**ICF_3_	− 7.82	− 2.20	− 14.33	16.35	2.02	− 7.64	− 6.80
[Pd(NCI⋅⋅⋅NH_3_)_3_(NCI)]^2+^**|**(NH_3_)	− 26.74	− 2.74	− 41.52	45.96	4.44	− 28.44	− 25.03
[Pd(NCI⋅⋅⋅NH_3_)_3_]^2+^**|**(NCI⋅⋅⋅NH_3_)	− 67.78	− 4.98	− 95.35	97.36	2.01	− 64.81	− 39.60
[Pd(NCI)_3_]^2+^**|**(NCI)	− 62.52	− 4.93	− 82.49	92.36	9.87	− 67.46	− 39.99

### Tuning the strength of halogen bonds

**Fig. 9:**
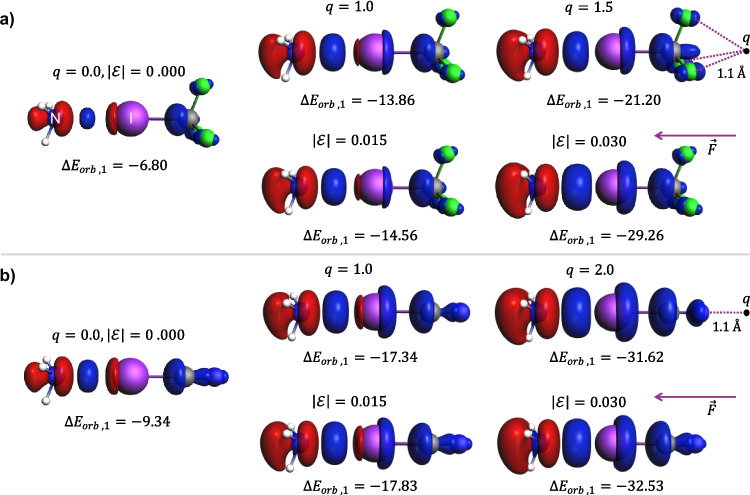
The dominant NOCV-pair contribution to the deformation density ($\Delta {\rho }_{1}$) for the N–I bond in NH_3_–ICF_3_ (**a**) and NH_3_–ICN (**b**) in the presence of a point charge *q* at a distance of 1.1 Å to the terminal atom(s) and a homogeneous external electric field of various strength ($\left|\mathcal{E}\right|$, in a.u.) along with the corresponding interaction energies ($\Delta {E}_{orb,1}$, in kcal/mol). The blue/red regions of $\Delta \rho$ indicate the accumulation/depletion of the electron density, and the purple arrows represent the direction of the electric field. For the complete set of the total interaction energy components, see Tables 2 and 3

To extend the comparison between halogen bonds and the dative bond, we considered models in which the strength of the halogen bond can be tuned. The idea was to increase the strength of the halogen bond by increasing “the depth” of the σ-hole. We anticipated that this can be achieved by applying an external electric field. For both, NH_3_⋅⋅⋅ICF_3_ and NH_3_⋅⋅⋅ICN systems, we first used increasing positive point-charge located at the extension of the I-CF_3_/I-CN bonds (Fig. 9). We assumed that such location of the positive charge should result in pulling the electrons from I-CF_3_/I-CN bonds and increasing “the depth” of the σ-hole. In a sequence of single-point calculations, the value of the charge was systematically changed, increasing from 0.0 to 2.0 e. Certainly, the values of the charge and the exact position of the point charge are arbitrary. To remove the latter arbitrariness, we also tested the effect of the external electric field by considering explicitly the electric field vector (see Fig. 9). The calculated ETS energy components are collected in Tables 2 and 3, and the NOCV-deformation density components are shown in Fig. 9. The results indeed show that polarization of ICF_3_/ICN by electric field can increase the strength of halogen bonding and increase the orbital interaction energy to an order of magnitude comparable to that of dative-covalent bonds. However, when going from non-covalent to dative-covalent regime (concerning energy), the qualitative picture of the major NOCV-based contribution Δρ_1_ remains similar (Fig. 9). In particular, the overall shape of the contours in Fig. 9a and b remains practically the same. Some increase in the accumulation of the electron density between N and I atoms can be observed due to an increase in the electric field, but this effect is not as dramatic as could be expected from a relatively large increase in the orbital interaction energy.

**Table 2:** Changes in the total interaction energy components and the dominant NOCV-pair contribution to the orbital interaction energy $\Delta {E}_{orb,1}$ (in kcal/mol) for the N–I bond in NH_3_⋅⋅⋅ICF_3_ in the presence of a point charge *q* at a distance of 1.1 Å from each of the fluorine atoms oriented along the N–I bond or homogeneous external electric field of various strengths $\left|\mathcal{E}\right|$ (in a.u.) oriented along the N–I bond and directed from the I to N. Single point calculations

Point charge/electric field	Δ*E*_int_	Δ*E*_disp_	Δ*E*_elstat_	Δ*E*_Pauli_	Δ*E*_steric_	Δ*E*_orb_	Δ*E*_orb,1_
-	− 7.82	− 2.20	− 14.33	16.35	2.02	− 7.64	− 6.80
*q*							
0.5	− 13.53	− 2.20	− 15.88	15.29	− 0.59	− 10.74	− 9.66
1.0	− 20.84	− 2.20	− 17.56	14.45	− 3.11	− 15.53	− 13.86
1.5	− 31.77	− 2.20	− 19.35	13.76	− 5.59	− 23.97	− 21.20
$$\left|\mathcal{E}\right|$$							
0.005	− 10.92	− 2.20	− 14.92	15.98	1.06	− 9.78	− 8.75
0.010	− 14.75	− 2.20	− 15.53	15.66	0.13	− 12.68	− 11.31
0.015	− 19.38	− 2.20	− 16.17	15.39	− 0.78	− 16.40	− 14.56
0.020	− 24.89	− 2.20	− 16.82	15.16	− 1.66	− 21.03	− 18.58
0.025	− 31.39	− 2.20	− 17.49	14.96	− 2.53	− 26.66	− 23.45
0.030	− 38.95	− 2.20	− 18.17	14.78	− 3.39	− 33.36	− 29.26

**Table 3:** Changes in the total interaction energy components and the dominant NOCV-pair contribution to the orbital interaction energy $\Delta {E}_{orb,1}$ (in kcal/mol) for the N–I bond in NH_3_⋅⋅⋅ICN in the presence of a point charge *q* at a distance of 1.1 Å from the ICN nitrogen atom oriented along the N–I bond homogeneous external electric field of various strengths $\left|\mathcal{E}\right|$ (in a.u.) oriented along the N–I bond and directed from the I to N. Single point calculations

Parameter	Δ*E*_int_	Δ*E*_disp_	Δ*E*_elstat_	Δ*E*_Pauli_	Δ*E*_steric_	Δ*E*_orb_	Δ*E*_orb,1_
0.000	− 10.77	− 2.38	− 20.51	22.64	2.13	− 10.52	− 9.34
*q*							
0.50	− 17.80	− 2.38	− 22.56	21.39	− 1.17	− 14.25	− 12.74
1.00	− 25.91	− 2.38	− 24.77	20.64	− 4.13	− 19.40	− 17.34
1.50	− 35.65	− 2.38	− 27.15	20.17	− 6.98	− 26.28	− 23.42
2.00	− 47.73	− 2.38	− 29.68	19.91	− 9.77	− 35.57	− 31.62
$$\left|\mathcal{E}\right|$$							
0.005	− 14.23	− 2.38	− 21.13	22.33	1.19	− 13.04	− 11.58
0.010	− 18.38	− 2.38	− 21.78	22.06	0.27	− 16.27	− 14.39
0.015	− 23.28	− 2.38	− 22.46	21.83	− 0.63	− 20.27	− 17.83
0.020	− 29.02	− 2.38	− 23.16	21.64	− 1.52	− 25.11	− 21.95
0.025	− 35.65	− 2.38	− 23.88	21.47	− 2.41	− 30.85	− 26.83
0.030	− 43.24	− 2.38	− 24.63	21.34	− 3.29	− 37.57	− 32.53

**Fig. 10:**
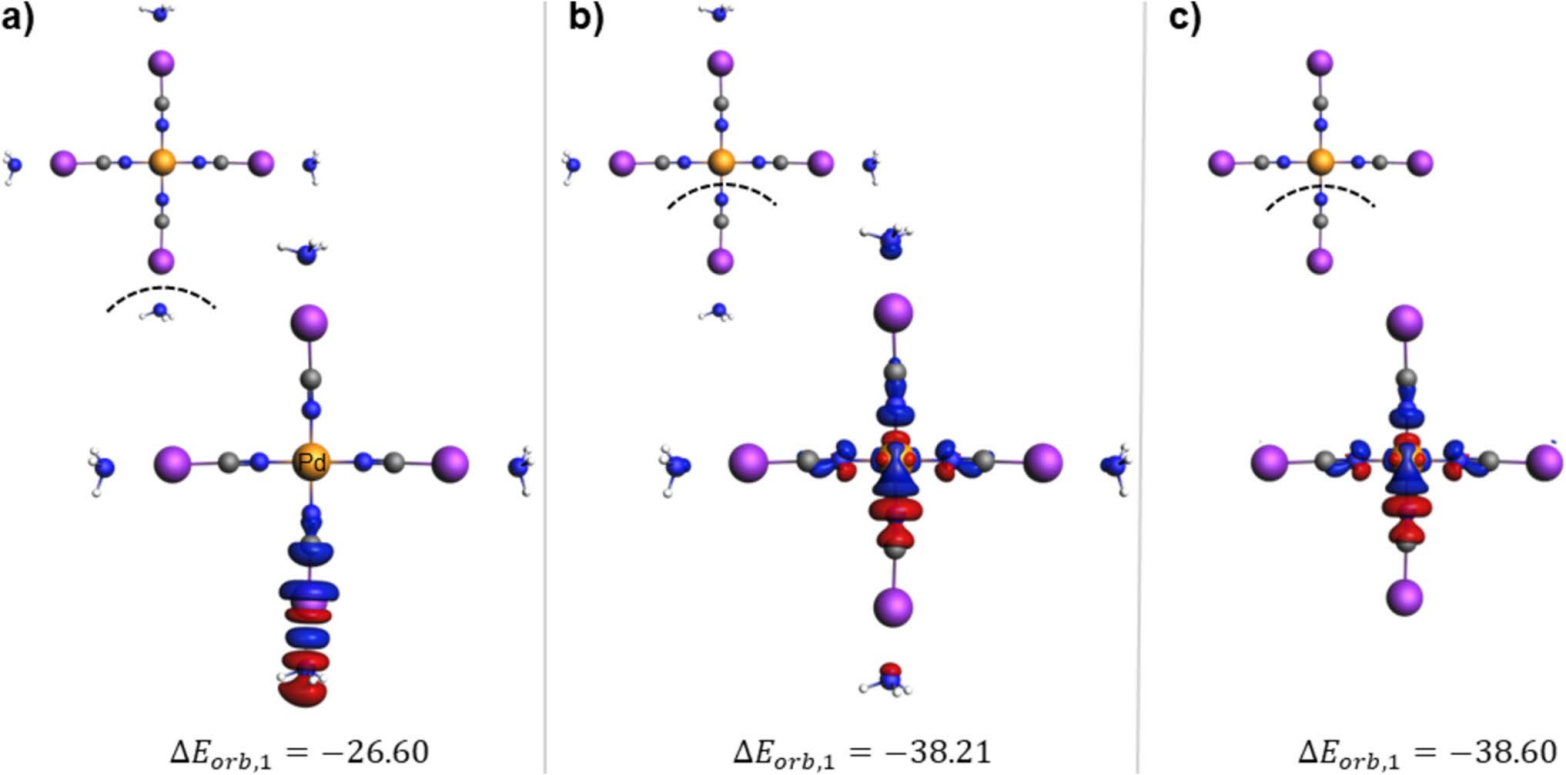
ETS-NOCV fragmentation patterns along with isosurfaces (|0.002| a.u.) of the dominant deformation density contributions with the corresponding orbital interaction energies $\Delta {E}_{orb,1}$ (in kcal/mol) describing the N⋅⋅⋅I interaction in [Pd(NCI⋅⋅⋅NH_3_)_4_]^2+^ complex (**a**) and the N–Pd bond in [Pd(NCI⋅⋅⋅NH_3_)_4_]^2+^ (**b**) and [Pd(NCI)_4_]^2+^ (**c**). The blue/red regions of $\Delta \rho$ indicate the accumulation/depletion of the electron density

To construct a more realistic molecular model in which the strength of halogen bond increases, we consider the binding of NH_3_⋅⋅⋅ICN to a transition metal, in the [Pd(NCI⋅⋅⋅NH_3_)_4_]^2+^ complex. In Fig. 10a, the ETS-NOCV results for halogen bonding in this system are summarized by considering an ammonia molecule and the rest of the complex as two fragments. To obtain a more complete picture in parts b and c of Fig. 10, we present the results of the analysis performed for the bond between Pd and the ligand with and without the halogen bond, i.e., in [Pd(NCI⋅⋅⋅NH_3_)_4_]^2+^ and [Pd(NCI)_4_]^2+^. The ETS-NOCV energy components are included in Table 1. The results show that the presence of the metal ion leads to the strengthening of the NCI⋅⋅⋅NH_3_ halogen bond, indeed. This is reflected by the greater magnitude of the total interaction energy compared to that of the isolated ligand ($\Delta {E}_{int}=-26.74$ vs. $-10.78$ kcal/mol, see Table 1). This stabilization is primarily due to electrostatic interactions ($\Delta {E}_{elstat}=-41.52$ vs. $-20.51$ kcal/mol) which are much larger than the corresponding values of the orbital interaction energy ($\Delta {E}_{orb}=-28.44$ vs. $-10.52$ kcal/mol). Noticeably, the leading NOCV channel Δρ_1_ is qualitatively similar for NCI⋅⋅⋅NH_3_ and Pd-assisted [(NCI⋅⋅⋅NH_3_)_3_Pd]-NCI⋅⋅⋅NH_3_ (Fig. 10a vs Fig. 9b). Finally, the presence of the electron-donor (ammonia) influences only slightly (by ca. $5$ kcal/mol) the strength of the DCD type of N–Pd bond (Table 1, Fig. 10b, c).

### ΔMEP picture of halogen bonding in NH_3_⋅⋅⋅ICF_3_ and NH_3_⋅⋅⋅ICN

**Fig. 11:**
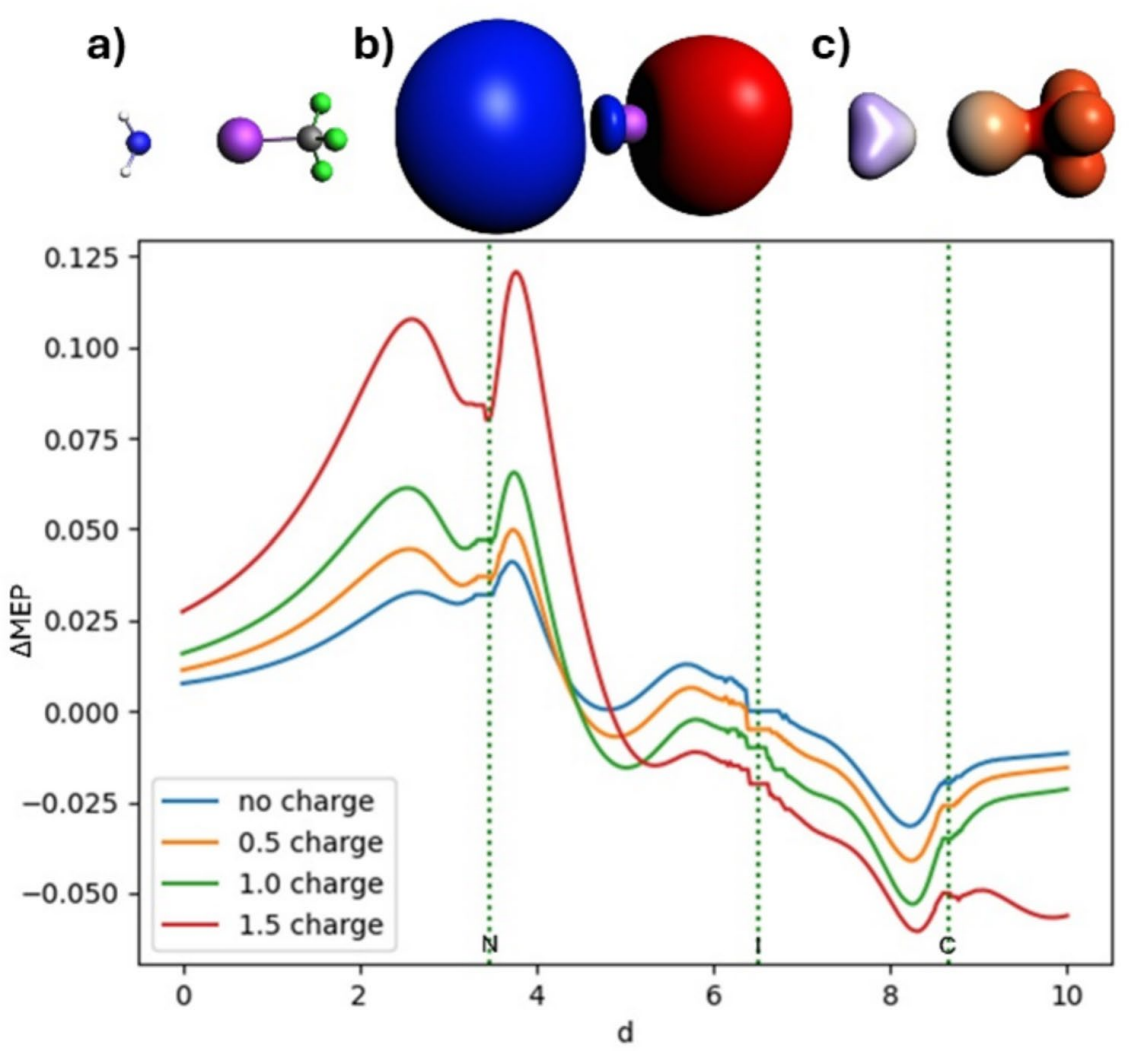
Analysis of ΔMEP for the ammonia-trifluoroiodomethane complex: In the top row, the molecule (**a**), the ΔMEP contour with a value of 0.005 (**b**), and the ΔMEP projected onto an isodensity contour of electron density with a value of 0.01 (**c**) are shown. The positive/negative ΔMEP values are indicated in blue/red. The graph shows four curves, representing MEP deformation with no point charge, and with positive point charges of 0.5, 1, and 1.5 units on the bond axis. The dotted green lines indicate the positions of the atomic nuclei

**Fig. 12:**
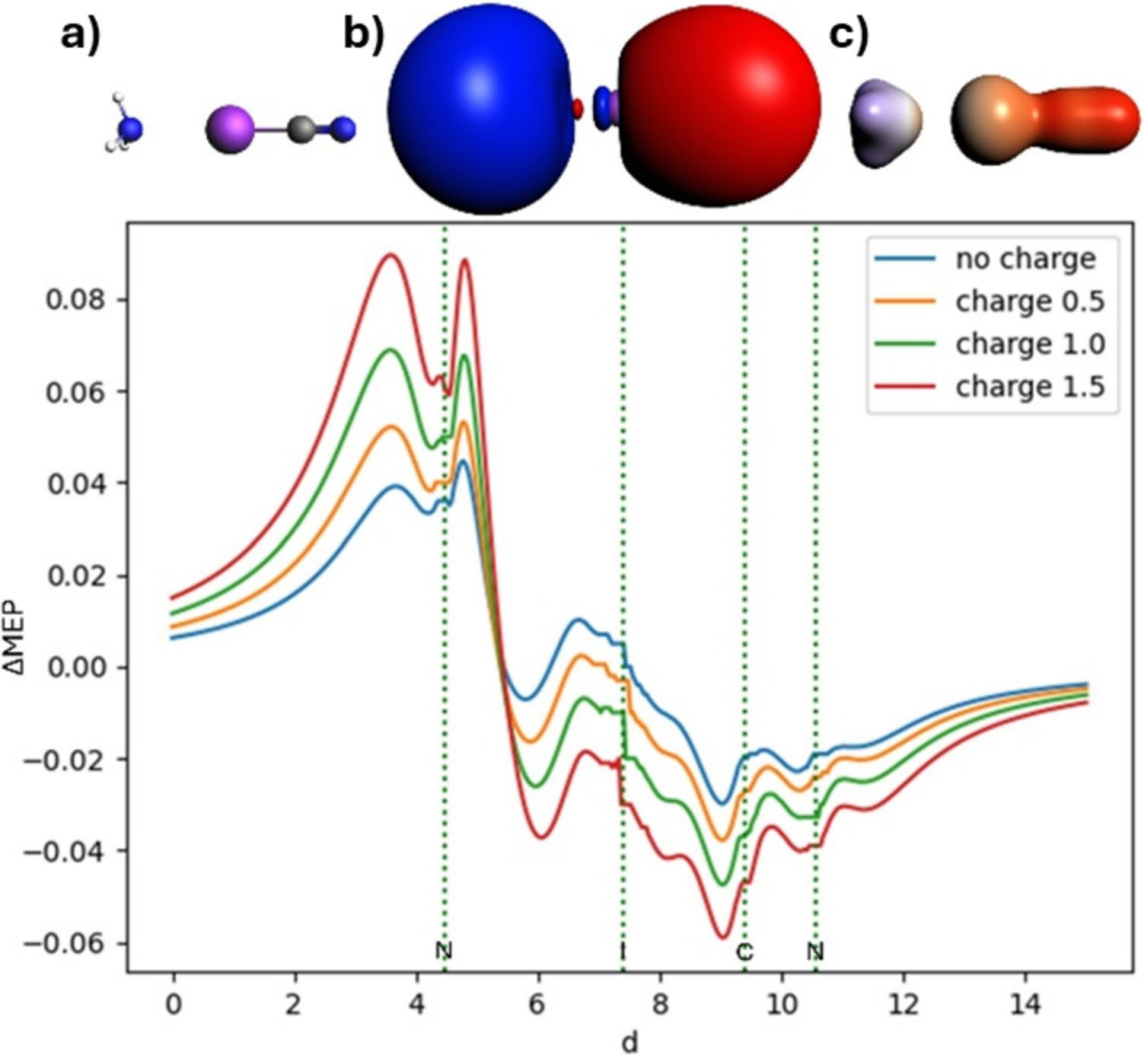
Analysis of ΔMEP for the ammonia-iodine cyanide complex: In the top row, the molecule (**a**), the ΔMEP contour with a value of 0.005 (**b**), and the ΔMEP projected onto an isodensity contour of electron density with a value of 0.01 (**c**) are shown. The positive/negative ΔMEP values are indicated in blue/red. The graph shows four curves, representing MEP deformation with no point charge, and with positive point charges of 0.5, 1, and 1.5 units on the bond axis. The dotted green lines indicate the positions of the atomic nuclei

In Figs. 11 and 12, we present the ΔMEP picture for NH_3_⋅⋅⋅ICF_3_ and NH_3_⋅⋅⋅ICN, respectively. Like in the ETS-NOCV analysis presented above, we consider those systems without and with an electric field modeled by increasing point charge. For both systems, the ΔMEP picture is dominated by the polarization of NH_3_ and ICF_3_ fragments, with positive values “on the NH_3_ side” and negative values “on the ICF_3_/ICN side.” Along the N-I bond axis, two maxima and a minimum appear between the atoms. The blue curve, representing the molecule without an additional point charge, shows the smallest variations in electrostatic potential. As the value of the positive point charge increases from 0.5 to 1.5, these differences become more pronounced, indicating that the bond strengthens. The minima on the N-I bond axis have values of − 0.0004, − 0.007, − 0.015, and − 0.014 a.u. for NH_3_⋅⋅⋅ICF_3_ with point charges ranging from 0 to 1.5. For NH_3_⋅⋅⋅ICN, the values of the corresponding minima are − 0.005, − 0.016, − 0.026, and − 0.037 a.u. Thus, an increase in the electron density accumulation “in the halogen bond area” is reflected by the increased magnitude of the negative ΔMEP; this is consistent with the ETS-NOCV picture. However, the changes in ΔMEP due to the electric field in the halogen bond area are not as small as the changes observed “in the fragments areas,” indicating their polarization. With an increase in the electric field, the picture becomes qualitatively more similar to that of the dative bond in borazane, but the depth of the minima is incomparably smaller for halogen bonding. This clearly indicates that the polarization of the fragments is much more important than “the covalent” contribution.

**Fig. 13:**
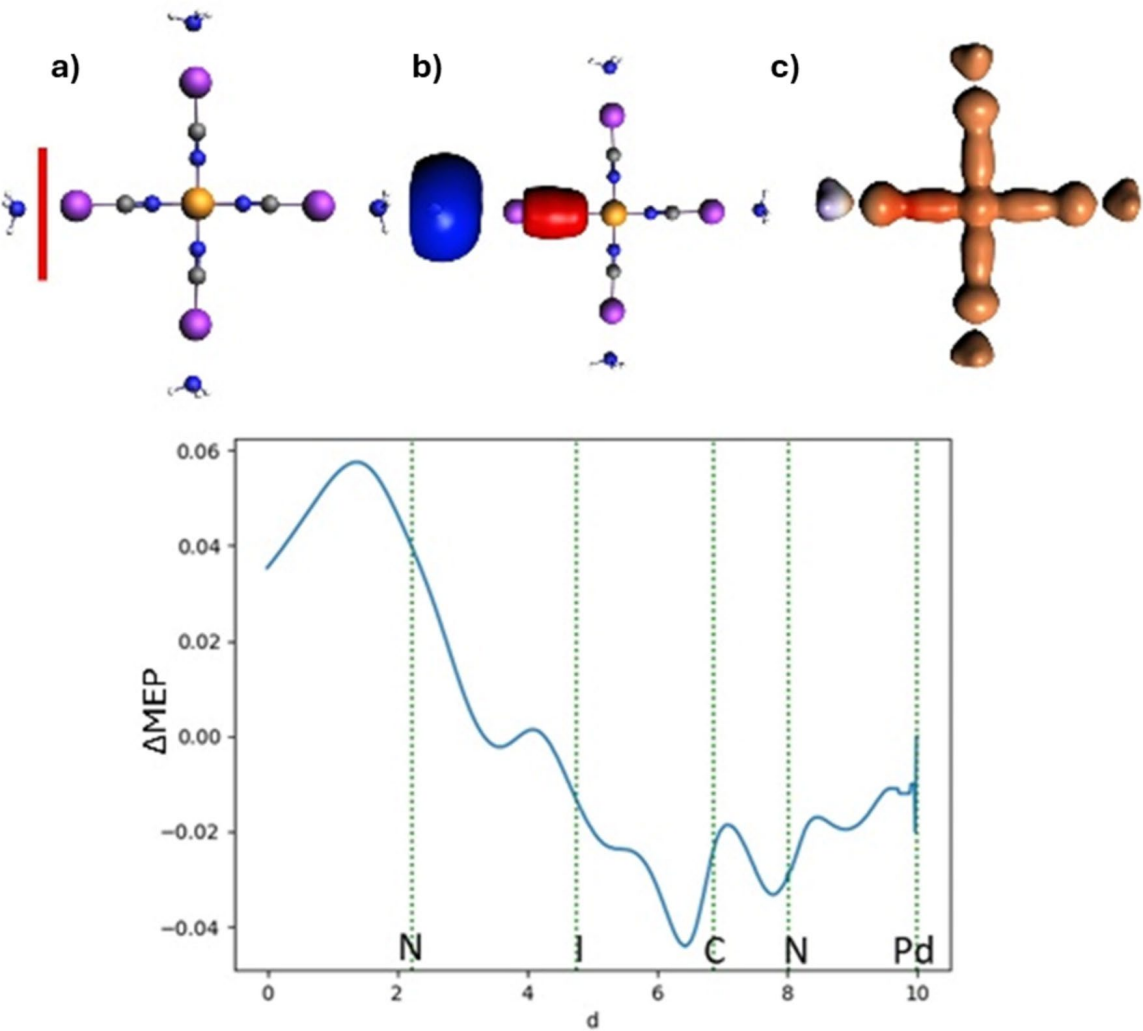
Analysis of ΔMEP for [Pd(NCI⋅⋅⋅NH_3_)_4_]^2+^: In the top row, the molecule (**a**), the ΔMEP contour with a value of 0.02 (**b**), and the ΔMEP projected onto an isodensity contour of electron density with a value of 0.01 (**c**) are shown. The positive/negative ΔMEP values are indicated in blue/red. The graph depicts the ΔMEP value along the bond axis of the molecule. The dotted green lines indicate the positions of the atomic nuclei

Finally, the ΔMEP analysis for the halogen bond in [Pd(NCI⋅⋅⋅NH_3_)_4_]^2+^ is presented in Fig. 13. The ΔMEP plots show a similar interaction pattern to that in NH_3_-ICN (Fig. 12); though this time, the interaction was influenced by the Pd^2+^ cation instead of by a point charge. The ΔMEP plot along the N-I bond axis in the presence of the Pd^2+^ cation indicates a more pronounced polarization than that of the isolated NH_3_–-ICN system. The presence of the Pd^2+^ cation enhances the strength of halogen bonding. However, as in the case of point charges, the depth of the ΔMEP minima is much smaller (almost by an order of magnitude) for halogen bonding than for dative bond in borazane.

## Concluding remarks

One of the important advantages of ETS-NOCV analysis is that this approach can be easily applied in the analysis of various types of interactions between molecular fragments. In this contribution, we presented the interpretation of ETS-NOCV for typical covalent and dative-covalent chemical bonds, as well as for halogen bonds. We considered tuning the strength of halogen bonding first by applying an electric field (modeled by the point charges or the electric field vector) and then by constructing a model transition-metal complex in which the strength of halogen bonding was enhanced. For all the systems, the ETS-NOCV picture was supplemented by the analysis of the deformation in molecular electrostatic potential (ΔMEP).

The results demonstrate important characteristic features of the analysis based on NOCV. First, this approach is based on pairs of orbitals with antibonding and bonding character; thus, it allows us to “extract” a “diatomic-like” picture of chemical bonding. Here, however, the antibonding and bonding NOCV usually have fractional occupations in both, the molecule and the promolecule. Furthermore, the intrinsic feature of the analysis based on NOCV is the DCD-model-like picture; the NOCV pair-contributions to the deformation density often correspond to donation (A→B) and back-donation (A←B) of electron density between the fragments.

The results for halogen bonding demonstrate that it is possible to tune their strength by an electric field in the molecular environment, e.g., by the presence of transition metal. The halogen bond energy can reach the order of magnitude typical of dative-covalent bond. However, the results also show that the nature of halogen bonds still differs from that of dative-covalent interactions, as the accumulation of electron density between fragments is much lower in the former case. The main effect of the electric field is an increase in the polarization of the fragments. This is clearly manifested by the deformation in the MEP. Due to the long-range character of the electrostatic potential, the analysis of ΔMEP leads to a clearer indication of charge flows and polarization of the fragments than does the analysis of deformation density. Therefore, the analysis of ΔMEP can be a valuable supplement to ETS-NOCV analysis.

## Data Availability

Data is provided within the manuscript or supplementary information files. Competing Interests The authors declare no competing interests.
